# A Bivalent Protease-Activated Receptor-Derived Peptide Mimics Neuronal Anti-Apoptotic Activity of Activated Protein C

**DOI:** 10.3390/bioengineering12090899

**Published:** 2025-08-22

**Authors:** Abhay Sagare, Youbin Kim, Kassandra Kisler, Ruslan Rust, William J. Mack, José A. Fernández, Berislav V. Zlokovic, John H. Griffin

**Affiliations:** 1 Department of Physiology and Neuroscience, Keck School of Medicine, University of Southern California, Los Angeles, CA 90033, USA; sagare@usc.edu (A.S.); youbin218@gmail.com (Y.K.); kislerel@usc.edu (K.K.); rrust@usc.edu (R.R.); berislav.zlokovic@gmail.com (B.V.Z.); 2 Zilkha Neurogenetic Institute, University of Southern California, Los Angeles, CA 90033, USA; 3 Department of Neurological Surgery, Keck School of Medicine, University of Southern California, Los Angeles, CA 90033, USA; william.mack@med.usc.edu; 4 Department of Translational Medicine, The Scripps Research Institute, 10550 N. Torrey Pines Road, IMM316, La Jolla, CA 92037, USA; jfernand@scripps.edu

**Keywords:** neuron, apoptosis, protein C, protease-activated receptor, G-protein coupled receptor

## Abstract

Activated protein C (APC) exerts anticoagulant and cytoprotective cell signaling activities. APC’s cell signaling requires protease-activated receptor (PAR) PAR1 and PAR3, and APC’s PAR cleavages generate peptides capable of agonizing biased G-protein coupled receptor (GPCR) cytoprotective signaling, resulting in anti-inflammatory and anti-apoptotic activities and endothelial barrier stabilization. The PAR-sequence-derived 34-residue “G10 peptide” comprising PAR1 residues 47–55 covalently attached by a 10-glycine linker to PAR3 residues 51–65 is an orthosteric/allosteric bivalent GPCR agonist that potently mimics APC’s anti-inflammatory activity and endothelial barrier stabilization activity. The objective of this study was to determine whether the G10 peptide mimics APC’s anti-apoptotic activity using cultured murine neurons challenged by N-methyl-d-aspartate that provokes neuronal apoptosis. In these new studies, the G10 peptide mimicked APC’s anti-apoptotic activity. Thus, the PAR-derived 34-residue G10 peptide mimics APC’s three major cytoprotective activities, namely anti-inflammatory and anti-apoptotic activities and endothelial barrier stabilization. Peptides that agonize GPCRs provide promising and currently approved drugs; e.g., semaglutide and tirzepatide that contain 31 and 39 amino acid residues, respectively. Thus, this new study adds to the rationale for pursuing further studies of the G10 peptide for potential therapeutic value for multiple pathologies where APC or signaling-selective APC variants are therapeutic in preclinical animal studies.

## 1. Introduction

Plasma-derived activated protein C (APC), a serine protease, demonstrated anti-inflammatory, antithrombotic, and neuroprotective effects in a murine model of focal ischemic stroke [1]. Following that 2001 report, extensive studies showed that APC exerts not only anticoagulant activity but also multiple cellular cytoprotective activities, including anti-apoptotic and anti-inflammatory activities and endothelial barrier-stabilizing activity, and that APC’s cell signaling primarily involves cleavage and biased activation of protease-activated receptor (PAR)1 and PAR3 (see reviews [2,3]). The human and murine genomes contain 4 G protein coupled receptors (GPCRs), designated PARs, which are activated by proteolytic cleavage of the extracellular N-terminal tail, which creates a new N-terminal tail whose terminal amino acid sequence serves as a tethered agonist for initiating signaling [2,3]. Biased activation of a GPCR indicates that the same GPCR can trigger very different signaling responses when activated by two different agonists; e.g., when PAR1 is cleaved at different sites by either thrombin or by APC [2,3]. Peptides derived from the extracellular N-terminal tails of PAR1 or PAR3 can also initiate cell signaling that mimics APC’s cell signaling that results in endothelial barrier stabilization and anti-inflammatory effects [4,5,6,7]. Recently, we showed that a bivalent PAR-derived peptide, designated the G10 peptide which contains covalently linked PAR1-derived and PAR3-derived peptides, potently mimics APC’s anti-inflammatory cytoprotection and that this bivalent peptide is far more potent than either the single PAR1-derived or PAR3-derived peptide alone [7].

Many approved drugs as well as drugs currently in clinical trials agonize or antagonize GPCRs [8]. Several peptide drugs that agonize GPCRs are very high profile, e.g., semaglutide and tirzepatide that contain 31 and 39 amino acid residues, respectively [8,9,10]. The demonstration that the bivalent G10, a 34-residue peptide, is proven to function as a PAR agonist that mimics two of APC’s major cytoprotective activities, namely endothelial stabilization and anti-inflammatory activity [7], stimulated the current study for G10 based on the hypothesis that G10 may mimic one of APC’s diverse neuroprotective actions, specifically APC’s neuronal anti-apoptotic activity.

Ischemia/reperfusion injury and hypoxia are complex and can lead to neuronal cell death as observed in ischemic stroke. Neuronal excitotoxicity, i.e., toxic overactivation of neurons, induced by N-methyl-D-aspartate (NMDA) can lead to neuronal apoptosis that is dependent on NMDA receptor (NMDAR) activation [11]. In this report, we extend studies of the bivalent PAR-derived G10 peptide to show that it mimics APC’s ability to inhibit NMDA-induced neuronal apoptosis of cultured neurons [12]. Primary mouse neuron cell culture models have been widely used for the screening of neuroprotective agents including drugs blocking neuronal excitotoxicity and loss of neurons [13,14]. In the studies described here, a similar neuron cell culture model is used to compare the anti-apoptotic activity of the G10 peptide to that of APC after NMDA challenge.

## 2. Materials and Methods

### 2.1. Cell Culture and Immunocytochemistry Reagents

The following cell culture reagents were purchased from Thermo Fisher Scientific USA (Waltham, MA, USA): B-27^TM^ Plus Neuronal Culture System (B-27 Plus Supplement (50X) and Neurobasal Plus Medium, catalog number A3653401), GlutaMAX^TM^ supplement (catalog number 35050061), Dulbecco’s Modified Eagle Medium (DMEM) (catalog number 31053-028), Ca^2+^ and Mg^2+^-free Earle’s Balanced Salt Solution (EBSS, catalog number 14155-063), Ca^2+^ and Mg^2+^-containing EBSS (catalog number 24010-043), Phosphate-buffered saline (PBS) (catalog number 10-010-023), Penicillin–Streptomycin (catalog number 15140122), poly-D-lysine (catalog number A38904-01), trypsin–EDTA (0.25%) phenol red (catalog number 25200056), and trypan blue (catalog number T10282). Poly-D-lysine coated 12 mm coverslips (catalog number GG-12-PDL) were purchased from Neuvitro Corporation, Camas, WA, USA).

For cell treatments, N-Methyl-D-aspartic acid, Hydrate (NMDA) (catalog number 454575) was purchased from EMD Millipore Corp., USA (Burlington, MA, USA). Human (h) plasma-derived activated protein C (hAPC) (catalog number APC) was purchased from Enzyme Research Laboratories (South Bend, IN, USA). The G10 bivalent peptide with the human PAR1 residues 47–55 sequence and PAR3 residues 51–65 sequence linked by 10 Gly residues (NPNDKYEPFGGGGGGGGGGFPFSALEGWTGATIT) was purchased from Bio-synthesis Inc, Lewiston, TX, USA.

The following reagents were purchased from Thermo Fisher Scientific USA: 16% formaldehyde solution (*w*/*v*), methanol-free (catalog number 28908), Hoechst 33258, pentahydrate (bis-benzimide) (catalog number H21491), donkey anti-rabbit Alexa Fluor^TM^ Plus 555 secondary antibody (catalog number A32794), FluoroPure^TM^ grade (catalog number H21491), ProLong^TM^ glass antifade mountant (catalog number P36980), and Click-iT^TM^ Plus terminal deoxynucleotidyl transferase dUTP nick labeling (TUNEL) assay (catalog number C10617). Triton^TM^ X-100 (catalog number X100) was purchased from MilliporeSigma. Normal donkey serum (catalog number 017-000-121) and donkey anti-chicken Alexa Fluor^®^ 488 secondary antibody were purchased from Jackson Immuno Research Laboratories, Inc (West Grove, Pennsylvania, USA). Anti-NeuN rabbit monoclonal antibody (catalog number 24307S) and an Alexa Fluor 555-conjugated cleaved caspase-3 rabbit monoclonal antibody (catalog Number 9604S) were purchased from Cell Signaling Technology (Danvers, MA, USA). Anti-MAP2 chicken polyclonal antibody was purchased from Abcam (catalog number ab5392). DAPI dihydrochloride (DNA-binding dye) (catalog number 268298) was purchased from MilliporeSigma (Burlington, MA, USA). Superfrost Plus^TM^ glass microslides (catalog number 48311-703) were purchased from VWR (Radnor, PA, USA).

### 2.2. Isolation and Culture of Primary Mouse Cortical Neurons

For the isolation and culture of primary mouse cortical neurons, timed-pregnant C57BL/6 mice (catalog number 000664) were purchased from the Jackson Laboratory for the culture of neurons from the cortices of embryos at day 16 of gestation. Briefly, after euthanasia, cortices from mouse embryos were collected and cut into pieces in DMEM with penicillin–streptomycin. After carefully aspirating the DMEM, the tissue was incubated in 0.25% trypsin–EDTA for 18 min at 37 °C in a cell culture incubator, followed by trituration with pipet tips and a glass pasture pipette. Dissociated live cells were counted with trypan blue, and the cell suspension was plated in 12-well tissue culture plates containing poly-D-lysine coated glass coverslips at a density of 6 × 10^5^ cells per well in serum-free B-27^TM^ Plus neurobasal medium. The primary cortical neuron cultures were maintained in a cell culture incubator at 37 °C for seven days, with media changes every two days.

### 2.3. Confirmation of Neuronal Cultures

After seven days in culture, three independent primary cortical neuron cultures on coverslips were washed with phosphate-buffered saline (PBS), fixed with 4% formaldehyde, and incubated in a blocking solution (5% donkey serum in PBS with 0.1% Triton X-100) for 1 h, followed by incubation with anti-NeuN and anti-MAP2 antibodies overnight (see reagents listed above). The cells were washed three times with a wash buffer (PBS with 0.1% Triton X-100) and incubated with anti-rabbit and anti-chicken secondary antibodies conjugated with Alexa Fluor^TM^ 555 and 488, respectively (1:250 dilution in blocking solution), for 1 h. After washing three times with the wash buffer, cell nuclei were counterstained with DAPI, and the cells were mounted onto microslides using ProLong^TM^ glass antifade mountant and imaged with a Nikon A1R inverted confocal microscope with 20X objective with 3x zoom in Galvano Scanning mode and Nikon NIS Elements Software v. 5.10.01. To calculate the percentage of NeuN positive cells out of the total number of nuclei in each image, the total DAPI-stained nuclei and NeuN-positive nuclei were counted in 3 random fields per replicate (60X magnification) using ImageJ v. 1.54f.

### 2.4. Induction and Analysis of Apoptosis and of Caspase-3 in Neuronal Cultures Treated with NMDA

To induce apoptosis with NMDA, after seven days of culture, the primary cortical neurons were exposed for 10 min to 300 µM NMDA + 5 µM glycine in Ca^2+^ and Mg^2+^-free EBSS as described earlier [13]. Cultures were washed once with Ca^2+^ and Mg^2+^-containing EBSS after exposure to NMDA and cultured with or without hAPC (100 nM) or G10 peptide (100 nM) in serum-free B-27^TM^ Plus neurobasal medium for 24 h. Cells were fixed with 4% formaldehyde in PBS for 15 min at room temperature. For detection of terminal deoxynucleotide transferase dUTP nick end labeling (TUNEL) after fixation of cells, the TUNEL assay was performed per the manufacturer’s instructions. Cell nuclei were counterstained with Hoechst 33258 (10 µg/mL in PBS) for 1 h at 37 °C, and the cells were mounted onto microslides using ProLong^TM^ glass antifade mountant.

For the cleaved or active form of caspase-3, fixed cells were incubated overnight with an Alexa Fluor 555-conjugated cleaved caspase-3 antibody. Cell nuclei were counterstained with Hoechst 33258 and mounted onto microslides using ProLong^TM^ glass antifade mountant. An independent observer blinded to the experimental conditions counted the number of TUNEL-positive and caspase-3-positive cells in 6 random fields per replicate (60X magnification) as the percentage of TUNEL-positive or caspase-3-positive cells out of the total number of nuclei using ImageJ.

Raw data that support findings of the apoptosis assays in this study are available from the senior authors upon reasonable request.

### 2.5. Western Blotting Analysis

The following reagents were purchased from Thermo Fisher Scientific USA: LDS sample buffer (catalog number NP0007), NuPAGE^TM^ 4–12% Bis-Tris midi gel (catalog number WG1402BX10), iBlot^TM^ 3 nitrocellulose transfer stacks midi NC (catalog number IB33001), HRP-conjugated donkey anti-rabbit secondary antibody (catalog number SA1-200), and Western blotting substrates (catalog numbers 32106 and 34096). Roche protease inhibitor (catalog number 04906837001) and phosphatase inhibitor (cocktail number 05892970001) cocktail were obtained from Millipore Sigma. Western blot blocking reagent (catalog number 13779-01) was purchased from Nacali Tesque, Inc (Kyoto, Japan). Blotting-grade nonfat dry milk (catalog number 1706404) and tris-buffered saline (TBS) (catalog number 1706435) were purchased from BIO-RAD. Tween^®^ 20 (catalog number P1379) was purchased from Sigma-Aldrich (Missouri, USA). A rabbit monoclonal anti-NR1 antibody (catalog number A11699) was purchased from Abclonal (Woburn, MA, USA). Rabbit anti-NR2A (catalog number 4205S), rabbit anti-NR2B (catalog number 4207S), and rabbit anti-β-actin (catalog number 4907L) antibodies and cell lysis buffer (10X, catalog number 9803) were purchased from Cell Signaling Technology, Inc. A rabbit polyclonal anti-NR2C antibody (catalog number PPS033) was purchased from R&D Systems (Minneapolis, MN, USA). Rabbit anti-NR2D (catalog number ab314664) and rabbit anti-NR3A (catalog number ab302516) antibodies were purchased from Abcam (Cambridge, UK).

After seven days of culture, isolated primary cortical neuronal cultures were lysed in cell lysis buffer containing Roche protease and phosphate inhibitor cocktail, and samples were prepared with LDS sample buffer. Next, 20 µg total protein was loaded per sample and separated on NuPAGE 4–12% Bis-Tris midi gel. After transfer to nitrocellulose membranes, membranes were blocked with Western blot blocking reagent for 1 h at room temperature and then incubated overnight at 4 °C with primary antibodies (listed above) diluted 1:1000 in blocking reagent. After washing three times with TBS containing 0.1% Tween 20 (TBST), membranes were incubated with HRP-conjugated anti-rabbit secondary antibodies diluted 1:3000 in TBST for 1 h at room temperature, washed three times with TBST. The membranes were then treated with Western blot chemiluminescent substrate for 5 min and imaged using a Cytiva Amersham Image Quant 800 system (Cytiva, Marlborough, MA, USA).

### 2.6. Statistical Analysis

Data are presented as mean ± standard deviation, with circles representing individual cultures. The Student’s *t*-test was used to determine statistically significant differences; normality was determined using the Shapiro–Wilk test. A *p* < 0.05 was considered statistically significant. All the statistical analyses were performed using GraphPad Prism v. 10 (GraphPad software, Boston, MA, USA).

## 3. Results

### 3.1. Characterization of Cultured Neuronal Cells

**Figure 1:**
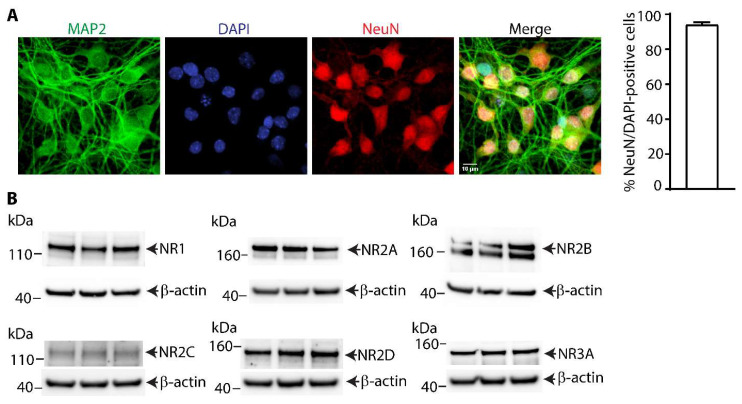
**Analysis of primary cultured mouse cortical neurons after seven days in culture.** (**A**) Neurons stained with MAP2 (green), nuclear stain DAPI (blue), and neuronal nuclear antigen (NeuN, red). DAPI-positive nuclei were on average 94% positive for NeuN neuronal marker in cultures (graph, mean ± SD, from three independent cultures). (**B**) Western blot analyses of NMDA receptor subunits NR1, NR2A, NR2B, NR2C, NR2D, and NR3A from three independent cultures with each lane representing an independent culture.

Immunostaining for neuron-specific microtubule-associated protein (MAP2) and neuronal nuclear antigen (NeuN) showed the presence of both neuronal markers in the mouse cortical neurons after seven days in culture (Figure 1A). Using the nuclear counterstain DAPI, approximately 94% of cells were found to be double-positive for NeuN and DAPI, indicating their primary neuronal origin. Three independent seven-day-old cultures were analyzed for the presence of NMDA receptor subunits using immunoblotting (Figure 1B). The NR1 and NR2 subunits of NMDA receptor tetrameric complexes [15] were expressed in these cultured neurons (Figure 1B). The NR1 subunit expression is essential for receptor formation, and its presence is crucial for NMDA receptor function, while the NR2 subunits evaluated (NR2A, NR2B, NR2C, and NR2D) contribute to the receptor’s distinct channel properties and gating. Importantly, the NR1 subunit and NR2B-containing NMDA receptors play a crucial role in NMDA-induced excitotoxicity in neurons in vitro. The NR2C and NR2D also participate in NMDA receptor function and can influence neuronal excitability. The NR3A subunit, which acts in a dominant negative manner to suppress receptor activity [16], was also expressed in the neuronal cultures (Figure 1B). Together these data indicate that these seven-day-old primary cortical neuronal cultures are suitable for studying NMDA-induced neuronal apoptosis.

### 3.2. Anti-Apoptotic Activity of G10 Peptide Mimics Anti-Apoptotic Activity of APC on NMDA-Challenged Neurons

**Figure 2:**
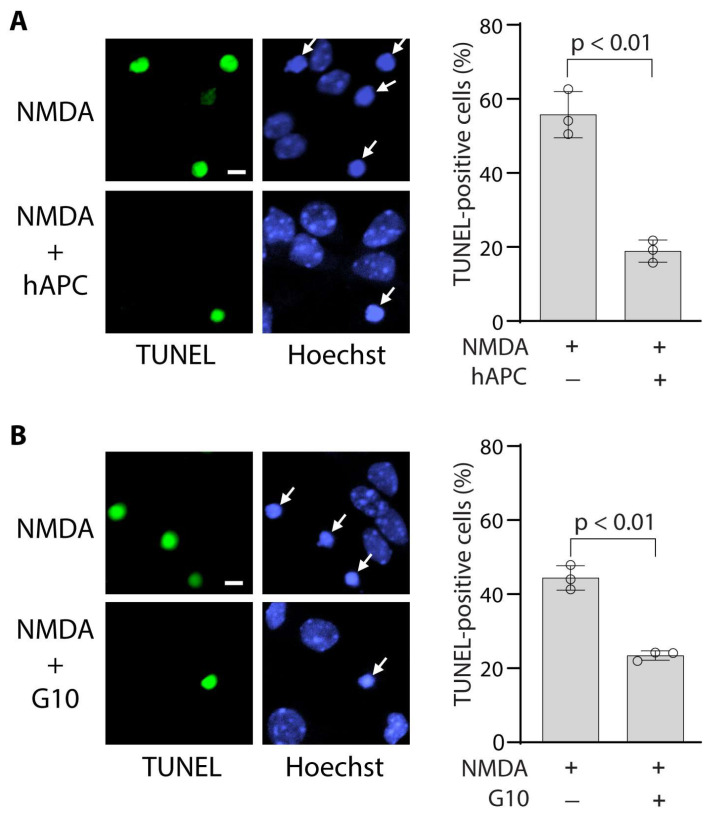
**APC and bivalent PAR-derived G10 peptide block NMDA-induced apoptosis in mouse cortical neurons.** (**A**,**B**) TUNEL-positive neurons (green) 24 h after NMDA in the absence or presence of human plasma-derived APC (hAPC, 100 nM) (**A**) or G10 peptide (100 nM) (**B**) and quantification of TUNEL-positive neurons. In (**A**,**B**), cell nuclei were visualized by Hoechst staining (blue). Arrows denote condensed nuclei. Bar = 5 µm. The number of TUNEL-positive neurons (**A**,**B**) was expressed as a percentage of total Hoechst-positive nuclei from three independent cultures and was determined from an average of six randomly chosen fields per culture. All analyses were performed by an observer blinded to the experimental conditions. Circles represent individual cultures. Bars are mean ± SD; *p* < 0.01 by two-tailed Student’s *t*-test.

Both hAPC (Figure 2A) and the PAR-derived G10 peptide (Figure 2B) blocked NMDA-induced apoptosis in cultures of mouse cortical neurons as shown by reduced numbers of TUNEL-positive cells, which were significantly reduced by 64% and 47%, respectively, at 24 h after NMDA treatment. This is consistent with previous findings showing that caspase-3 activity is induced by NMDA in seven-day-old cortical neuronal cultures [17].

**Figure 3:**
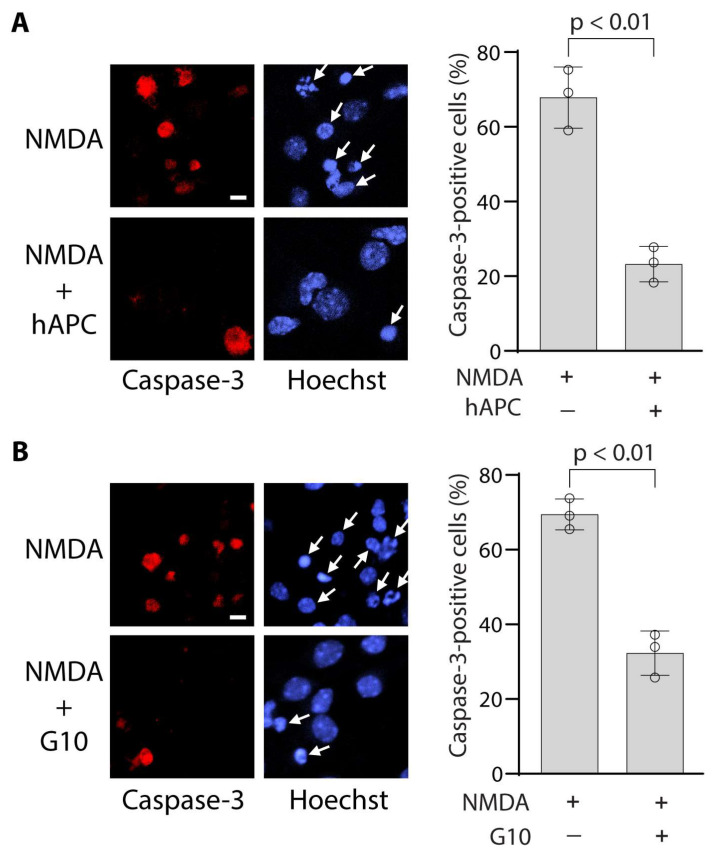
**Effects of APC and bivalent PAR-derived G10 peptide on caspase-3 in NMDA-treated mouse cortical neurons.** (**A**,**B**) Caspase-3-positive neurons (red) 24 h after NMDA in the absence or presence of hAPC (100 nM) (**A**) and or G10 peptide (100 nM) (**B**) and quantification of Caspase-3-positive neurons. In (**A**,**B**), cell nuclei were visualized by Hoechst staining (blue). Arrows denote condensed nuclei. Bar = 5 µm. The number of caspase-3-positive neurons (**A**,**B**) were expressed as a percentage of total Hoechst-positive nuclei from three independent cultures and was determined from an average of six randomly chosen fields per culture. All analyses were performed by an observer blinded to the experimental conditions. Circles represent individual cultures. Bars are mean ± SD; *p* < 0.01 by two-tailed Student’s *t*-test.

Figure 3 illustrates that approximately 70% of cells were caspase-3-positive 24 h after NMDA treatment in the control groups. Both hAPC (Figure 3A) and the PAR-derived G10 peptide (Figure 3B) significantly reduced the number of caspase-3-positive cells by 65% and 53%, respectively, at 24 h after NMDA treatment.

## 4. Discussion

The study here using cultured murine neurons shows that the PAR-derived, bivalent G10 peptide mimics APC’s neuronal anti-apoptotic action. Both APC and the G10 peptide reduced NMDA-induced cell death and also reduced levels of the pro-apoptotic intracellular protease, caspase-3. These findings confirm and expand previous studies which showed that APC and the signaling-selective APC mutant, 3K3A-APC, can provide neuronal anti-apoptotic activity [2,3,18,19]. The G10 peptide’s anti-apoptotic activity also extends previous data which showed that the short PAR1-derived 9-mer peptide comprising PAR1 residues 47–55 can reduce apoptosis of NMDA-challenged rat neurons [20]. In studies of PAR-derived peptides that mimic APC’s anti-inflammatory activity, the bivalent G10 34-mer peptide was much more potent than the PAR1-derived 47–55 9-mer [21]. Notably, when used here at 100 nM concentration for reducing NMDA-induced neuronal apoptosis, the G10 peptide was very active whereas an earlier study reported that the PAR1 9-mer peptide required 2 μM to 20 μM for robust activity [21], indicating that the G10 34-mer peptide was over an order of magnitude more potent than the PAR1 9-mer peptide alone. Excitotoxicity-induced neuronal death can be either NMDA receptor-dependent or NMDA receptor-independent [22]. Future studies of the bivalent G10 peptide’s neuronal protective activity against NMDA receptor-independent excitotoxicity may be relevant for further studies since excitotoxicity is thought to contribute to serious damage or death of neurons in many neurologic diseases; e.g., ischemic stroke and Alzheimer’s disease [22].

GPCR drug discovery and development is central to the translation of basic research to medical practice [23]. Peptides that agonize or antagonize GPCRs provide major promising and currently approved drugs; e.g., semaglutide and tirzepatide that contain 31 or 39 amino acid residues, respectively [3,7,8,9,10,21]. Previous findings for the G10 peptide [7,21] plus the new data reported here establish that the PAR-derived bivalent G10 34-residue peptide can agonize GPCRs and can mimic three of APC’s major cytoprotective activities, namely endothelial stabilization, anti-inflammatory activity, and anti-apoptotic activity. Consequently, the current available data imply that there may be significant translational value for the G10 peptide or similar PAR1:PAR3-derived bitopic polypeptides for a variety of pathologies where APC has provided beneficial therapy in a variety of preclinical animal studies [1,2,3]. G10 mimics APC’s ability to reduce mortality in a murine sepsis model [7] and to reduce pathology in a murine colitis model [24]. Extensive basic studies and preclinical injury studies of APC’s cytoprotective PAR1-biased signaling [4] have shown that signaling-selective APC mutants, e.g., 3K3A-APC lacking anticoagulant activity but retaining normal cytoprotective signaling, provide beneficial effects for sepsis, pneumonia, ischemia reperfusion injury in brain, heart and kidney, ocular inflammation, experimental autoimmune encephalitis, Alzheimer’s disease, amyotrophic lateral sclerosis, colitis, skin pathologies, and white matter stroke [2,3,25,26,27,28,29,30,31]. Now knowing that G10 mimics APC’s anti-apoptotic activity as well as APC’s anti-inflammatory activity and endothelial stabilization actions, it is very attractive to pursue future studies of the ability of G10 to provide therapeutic benefits for this long list of preclinical injuries.

## 5. Conclusions

Data here showing that the PAR-derived 34-residue G10 peptide mimics APC’s neuronal anti-apoptotic activity combined with previous knowledge establish that the GPCR-agonizing G10 peptide mimics APC’s three major cytoprotective activities, namely anti-inflammatory and anti-apoptotic activities and endothelial barrier stabilization. Because G10 mimics APC’s anti-apoptotic activity as well as its anti-inflammatory activity and endothelial stabilization actions, it is even more attractive to pursue future studies of the ability of G10 to provide therapeutic benefits for a number of preclinical injuries where APC has shown benefits.

## Data Availability

Raw data that support findings of the apoptosis assays in this study are available from the senior authors upon reasonable request.

## Abbreviations

APC	Activated protein
PAR	Protease-activated receptor
NMDA	N-methyl-D-aspartate
DMEM	Dulbecco’s Modified Eagle Medium
EBSS	Ca^2+^ and Mg^2+^-free Earle’s Balanced Salt Solution
TUNEL	Terminal deoxynucleotide transferase dUTP nick end labeling
NeuN	Neuronal nuclear antigen
